# Temporal Dynamics and Influential Factors of Taste and Odor Compounds in the Eastern Drinking Water Source of Chaohu Lake, China: A Comparative Analysis of Global Freshwaters

**DOI:** 10.3390/toxins16060264

**Published:** 2024-06-09

**Authors:** Lixia Shang, Fan Ke, Xiangen Xu, Muhua Feng, Wenchao Li

**Affiliations:** 1 CAS Key Laboratory of Marine Ecology and Environmental Sciences, Institute of Oceanology, Chinese Academy of Sciences, Qingdao 266071, China; 2 State Key Laboratory of Lake Science and Environment, Nanjing Institute of Geography and Limnology, Chinese Academy of Sciences, Nanjing 210008, China; fke@niglas.ac.cn (F.K.); wchli@niglas.ac.cn (W.L.); 3 Changzhou Academy of Environmental Science, Changzhou 213022, China; xgxuc@163.com

**Keywords:** harmful algal blooms (HABs), geosmin, 2-methyl isoborneol, *β*-cyclocitral, *β*-ionone

## Abstract

The escalating proliferation of cyanobacteria poses significant taste and odor (T/O) challenges, impacting freshwater ecosystems, public health, and water treatment costs. We examined monthly variations in four T/O compounds from September 2011 to August 2012 in Chaohu Lake’s eastern drinking water source (DECL). More importantly, we compared the reported T/O occurrence and the related factors in freshwater bodies worldwide. The assessment of T/O issues indicated a severe and widespread problem, with many cases surpassing odor threshold values. Remarkably, China reported the highest frequency and severity of odor-related problems. A temporal analysis revealed variations in odor occurrences within the same water body across different years, emphasizing the need to consider high values in all seasons for water safety. Globally, T/O issues were widespread, demanding attention to variations within the same water body and across different layers. Algae were crucial contributors to odor compounds, necessitating targeted interventions due to diverse odorant sources and properties. A correlation analysis alone lacked definitive answers, emphasizing the essential role of further validation, such as algae isolation. Nutrients are likely to have influenced the T/O, as GSM and MIB correlated positively with nitrate and ammonia nitrogen in DECL, resulting in proposed control recommendations. This study offers recommendations for freshwater ecosystem management and serves as a foundation for future research and management strategies to address T/O challenges.

## 1. Introduction

Over the past few decades, the frequency and intensity of harmful cyanobacterial blooms in freshwater environments worldwide have increased, primarily due to eutrophication and global warming [1,2,3,4,5]. In addition to their association with oxygen depletion and the production of cyanotoxins, cyanobacteria are linked to taste and odor (T/O) compounds [6,7,8,9,10,11,12]. Undesirable tastes and odors have been observed to manifest genotoxic and estrogenic effects on fish hepatocytes, thereby posing potential risks to human health through the consumption of aquatic products [13]. Furthermore, the presence of T/O compounds has prompted a series of public grievances, particularly concerning the acceptability of surface waters for recreational purposes and the quality and safety of drinking water [10,11,14,15]. The occurrence of T/O events in freshwater ecosystems has evolved into a global water issue, with numerous instances documented in studies conducted across China, Japan, Korea, North America, and Europe, thereby underscoring the widespread significance of this concern [16,17,18,19,20,21,22,23].

Two bicyclic terpenoid compounds, namely, geosmin (trans-1,10-dimethyl-trans-9-decalol, GSM) and 2-methylisoborneol (1,2,7,7-tetramethyl-exo-bicycloheptan-2-ol, MIB), which are renowned for their earthy and musty odors, represent the most frequently identified T/O substances in freshwater bodies globally [24,25,26,27,28,29]. Additional investigations have been conducted on alternative T/O compounds associated with cyanobacterial blooms, such as *β*-cyclocitral (CYC) and *β*-ionone (ION). These compounds, stemming from the degradation of *β*-carotene, exhibit distinctive odors reminiscent of wood, violets, tobacco, and flowers and have been scrutinized in certain water sources [30,31,32]. The simultaneous presence of multiple T/O compounds introduces complexity to water quality management and constitutes a significant concern in the context of natural and drinking water in several countries, including China [11,12,22,33,34,35].

Given the generally minuscule odor threshold concentrations (OTCs) of T/O compounds, specifically, 4, 15, 19.3, and 7 ng·L^−1^ for GSM, MIB, CYC, and ION, respectively [36], these compounds can be perceptible to humans at trace levels. Notably, China has incorporated GSM and MIB into their drinking water quality standards, establishing current standard values for both compounds at 10 ng·L^−1^ [37]. The resilience of these compounds to oxidation posed a substantial challenge for aquaculture and drinking water facilities in terms of effective monitoring and control [38,39]. The adoption of advanced treatment methods or a multifaceted approach to eradicate T/O compounds imposes significant financial burdens on water utilities. Therefore, formulating a strategy to prevent the generation of T/O compounds in source water is imperative. As a result, a nuanced comprehension of the foundational information concerning odorants and their interrelation with environmental conditions became imperative for the control and management of odor sources. Furthermore, in the pursuit of eradicating T/O compounds from freshwater systems, it is paramount to discern the temporal and spatial patterns of variation and pinpoint the pivotal factors that contribute to the emergence of odorous manifestations.

The dynamics and influencing factors of T/O compounds in aquatic systems have been extensively investigated [16,21,24,40,41,42,43,44,45]. The occurrence of T/O compounds in water bodies has been associated with diverse biotic and abiotic parameters, encompassing the phytoplankton community, water temperature, pH, and nutrient concentrations and/or ratios, etc. [22,28,42,44,46,47,48,49,50,51]. These studies provided valuable insights for a more nuanced comprehension of the origins and impacts of odors in aquatic environments. However, the relationships between T/O compounds and environmental factors are intricate. Moreover, numerous ambiguous ecological interactions between cyanobacteria and other organisms contribute to a substantial disparity observed among various water bodies. It would prove advantageous if the presence of odorants could be correlated with monitored environmental parameters, as such associations could potentially serve to estimate the presence of these T/O compounds in water.

Chaohu Lake (30°25′–31°43′ N, 117°16′–117°51′ E), positioned in the central region of Anhui Province, stands as the fifth-largest freshwater lake in China. Functioning as a pivotal regional fishery and tourism resource, it simultaneously serves as a critical potable water reservoir for approximately one million residents [52]. Nonetheless, it is also a recipient of domestic sewage and industrial effluents originating from the surrounding urban and rural areas. In the late 1980s, the exchange rate with the Yangtze River decelerated due to the establishment of the Chaohu Lake Gate, leading to an upsurge in nutrient inputs resulting from rapid agricultural intensification and industrial development in the basin [53]. Consequently, Chaohu Lake has been grappling with eutrophication since the 1980s [52]. Despite its eutrophic status, Chaohu Lake retains its pivotal role as a strategic water reserve due to its substantial storage capacity. However, specific areas within Chaohu Lake accumulate a notable quantity of cyanobacteria, and the issue of distinct odors has adversely impacted water quality, necessitating further investigation.

For the early detection of T/O incidents to effectively manage the drinking water sources and their respective treatment facilities in Chaohu Lake, this study aimed to (1) investigate the monthly dynamics of dissolved and particulate compounds from four typical T/O (GSM, MIB, CYC, and ION), (2) identify cyanobacteria in the eastern drinking water source of Chaohu Lake, (3) estimate the water quality variables influencing T/O contents, and (4) conduct a comprehensive comparative analysis and discussion of documented T/O incidents and their associated determinants in freshwater environments globally.

## 2. Results and Discussion

### 2.1. Seasonal Variations of the Dissolved and Particulate T/O Compounds in the Eastern Drinking Water Source of Chaohu Lake

**Figure 1:**
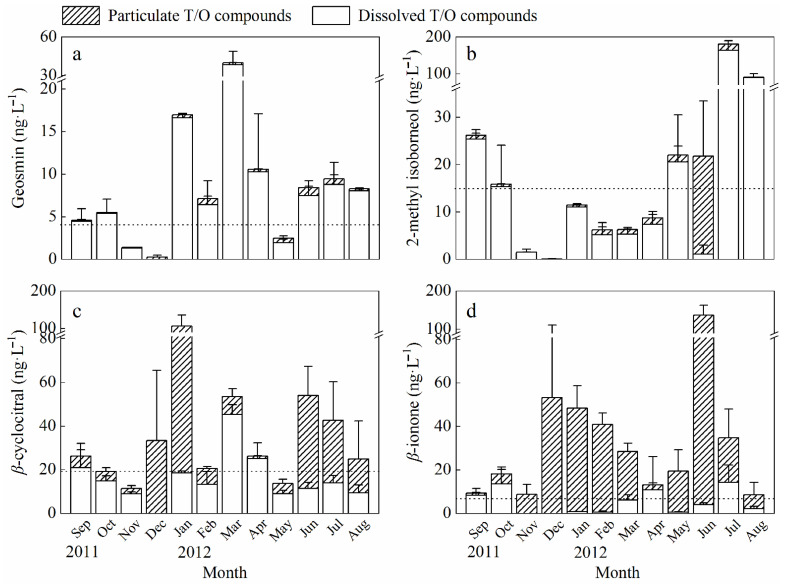
Seasonal variations of dissolved and particulate geosmin (**a**), 2-methyl isoborneol (**b**), *β*-cyclocitral (**c**), and *β*-ionone (**d**) in the eastern drinking water source of Chaohu Lake. Dotted lines indicate the OTCs of 4, 15, 19.3, and 7 ng·L^−1^ for geosmin, MIB, *β*-cyclocitral, and *β*-ionone, respectively, Watson (2004); Error bars indicate the standard deviation of three sampling sites.

In the eastern drinking water source of Chaohu Lake, the concentrations of dissolved and particulate T/O compounds displayed intricate seasonal variations (Figure 1). Geosmin exhibited its highest concentration in March (39.7 ± 10.6 ng·L^−1^), with particulate concentrations consistently below 2 ng·L^−1^. The concentrations of MIB were notably elevated from May to October, peaking at 180.5 ng·L^−1^ in July 2012. The maximum concentration of MIB in both the dissolved and particulate forms was recorded at 164.0 ng·L^−1^ (July 2012) and 20.7 ng·L^−1^ (June 2012), respectively. The average values for the total concentration of CYC in the investigated drinking water source exhibited three peaks in January 2012 (105.6 ng·L^−1^), March (53.5 ng·L^−1^), and June (54.0 ng·L^−1^), respectively, with both the dissolved and particle concentrations reaching their zenith in January 2012. Two distinct peaks in the total concentrations of ION were evident in December (53.3 ng·L^−1^) and June 2012 (136.6 ng·L^−1^), respectively. Although there is a limited body of literature specifically investigating T/O compounds in Chaohu Lake, it is evident that T/O issues are pronounced in this lake. This is manifested both by the reported maximum concentrations of four T/O compounds, with respective values of 1300 (GSM), 180.5 (MIB), 800 (ION), and 8300 ng·L^−1^, and the presence of significant olfactory problems in both the eastern and western regions of the lake (Refs. [11,22,33,42] and this study). The seasonal variations in the concentrations of T/O compounds within the eastern drinking water source of Chaohu Lake might be attributed to meteorological conditions, water temperature fluctuations, and biological activities [44,54].

Concerning the distribution between the dissolved and particulate forms, varied patterns were evident among different T/O compounds (Figure 1). Specifically, the four examined T/O compounds exhibited distribution patterns ranging from 11% to 91% outside cellular structures throughout the year, except for December, when no dissolved substance was detected. More than 79% of both GSM and MIB were found in the dissolved form, except for December, when the proportions were 0% for the former and 5% for the latter in June. Over the entire year, the average proportion of particulate CYC was 45%, comprising approximately 20% from September to November, 4–35% from February to May, and 62–100% in the remaining months. The majority of ION was observed within cellular structures, with an average particulate ratio of 71% throughout the year. Exceptions included 11–25% in April, September, and October and 59–78% in March, July, and August, with concentrations reaching 97–100% in the remaining months. Overall, GSM and MIB predominantly existed in dissolved form, while CYC and ION exhibited notable variations in their distribution between dissolved and particulate forms.

The aggregate concentrations of the four T/O compounds, encompassing both dissolved and particulate fractions, within the three designated sampling sites exhibited a range of 8.9–292.6 ng·L^−1^ (Figure 1). The concentrations of dissolved GSM, MIB, CYC, and ION surpassed their respective OTCs [36] in 75%, 42%, 25%, and 33% of the months, respectively. In terms of the overall concentrations, the proportions of GSM and MIB exceeding OTCs remained relatively constant, while those of CYC and ION increased to 75% and 100%, respectively. Hence, the concentrations of dissolved GSM, MIB, CYC, and ION surpassed their respective odor threshold concentrations in certain months, potentially impacting the perception and acceptability of water quality. The proportion of particulate forms, particularly observed in CYC and ION, demonstrated relatively higher proportions during specific months, indicating an inclination of these compounds to associate with particulate matter, possibly linked to biological activities in the water or organic matter in the sediment.

### 2.2. Factors Influencing the T/O Compound Variation in the Eastern Drinking Water Source of Chaohu Lake

Correlation coefficients were employed to assess the impact of biological, physicochemical, and organic indices on the temporal variability of T/O compounds in the eastern drinking water source of Chaohu Lake (Table 1). Notably, all four T/O compounds showed no significant correlation with cyanobacterial densities and Chl-*a* concentrations, suggesting independence from overall cyanobacterial abundance. However, specific associations emerged; MIB correlated positively with *Microcystis* spp., and ION showed a positive relationship with *Dolichospermum* spp. Both GSM and MIB displayed significant inverse correlations with *Dolichospermum* spp., indicating a nuanced interaction. In terms of physicochemical indices, MIB inversely correlated with dissolved oxygen and positively correlated with temperature, pH, and conductivity, suggesting sensitivity to these parameters. GSM exhibited a positive association with conductivity. Nutrient-wise, GSM and MIB correlated positively with nitrate and ammonia nitrogen, respectively, while ION had a negative correlation with total phosphorus, indicating nutrient influence. Regarding organic indices, MIB correlated positively with the potassium permanganate index and dissolved organic carbon, linking its concentration to water’s organic content.

**Figure 2:**
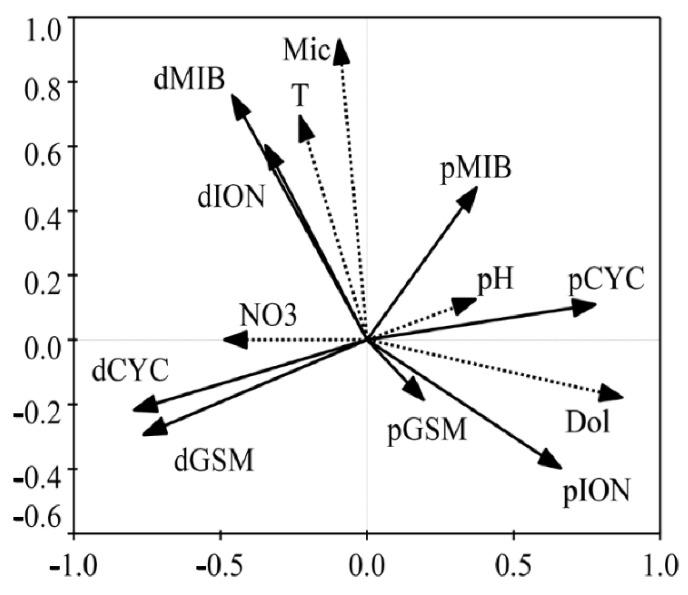
Redundancy analysis (RDA) biplots showed the concentrations of dissolved and particulate T/O compounds in relation to the density of *Microcystis* spp. (Mic) and *Dolichospermum* spp. (Dol) and the environmental factors, including water temperature (T), pH, dissolved oxygen, conductivity, TN, nitrate (NO_3_), nitrite, ammonia nitrogen, total phosphorus, and orthophosphate, in the eastern drinking water source of Chaohu Lake. d: dissolved T/O compounds; p: particulate T/O compounds.

The utilization of redundancy analysis systematically identified key environmental factors influencing the seasonal fluctuations of both dissolved and particulate T/O compounds. Among the fifteen examined variables, five, namely the densities of *Microcystis* spp. and *Dolichospermum* spp., water temperature, pH, and nitrate, significantly contributed to the variance of T/O compounds (*p* < 0.05) (Figure 2). The outcomes indicated a positive correlation between dissolved MIB and ION, both of which exhibited high associations with the cell density of *Microcystis* spp. and water temperature. Additionally, a positive correlation emerged between dissolved GSM and CYC, with the former positively and the latter negatively correlated with nitrate and pH, respectively. Moreover, particulate GSM and ION, along with particulate MIB and CYC, exhibited positive interrelations. All these particulate compounds were positively correlated with pH and the concentrations of *Dolichospermum* spp.

### 2.3. Relationships among T/O Compounds and Algal Toxins in the Eastern Drinking Water Source of Chaohu Lake

Considering the frequent co-occurrence of T/O compounds with algal toxins in eutrophic lakes experiencing cyanobacterial blooms [11,55], our investigation delved into the interrelationships among four T/O compounds and three prevalent microcystin (MC) variants, namely MC-LR, MC-RR, and MC-YR (Table 2). Positive correlations were observed among the three T/O compounds—GSM, CYC, and ION—suggesting a potential co-occurrence or similar influencing factors for these compounds. However, it is noteworthy that an inverse association was identified between GSM and MC-YR, indicating a contrasting relationship between these specific T/O and MC variants. Interestingly, no significant correlations were found between the other microcystin variants (MC-LR and MC-RR) and the examined T/O compounds. This suggests a complex and nuanced relationship between T/O compounds and specific microcystin variants, with potential variations in their production or dynamics. These findings contribute to our understanding of the interactions between T/O compounds and microcystins in eutrophic lakes during cyanobacterial blooms. The absence of correlations with certain microcystin variants suggests distinct regulatory mechanisms or factors influencing their dynamics.

### 2.4. Characteristics of the T/O Compounds in Freshwaters of the World

#### 2.4.1. Spatiotemporal Distribution of T/O Compounds in Freshwater Sources Worldwide

We compiled a review of the literature spanning from 1986 to 2023 that documented the presence of typical cyanobacteria-derived T/O compounds, namely GSM, MIB, CYC, and ION, in freshwater bodies worldwide (Table 3). The findings revealed prevalent T/O issues in raw water sourced from drinking water treatment plants, lakes, reservoirs, rivers, aqueducts, and fishponds, with manifestations reported in diverse regions, including China, Egypt, Germany, Italy, Japan, Korea, Spain, Switzerland, Thailand, the United States of America, and North America. These freshwater bodies exhibited variability in T/O types, concentrations, and the months of maximum occurrence.

GSM and MIB are two of the most prominent musty/earthy compounds detected in natural water systems, exhibiting significant concentrations that pose potential risks. GSM concentrations have reached alarmingly high levels, with a record of 7200 ng·L^−1^ reported in the YH Reservoir in Hebei Province, China, in July 2007 [26]. Similarly, MIB concentrations peaked at 5302.7 ng·L^−1^ in fishponds in Tianjin, China, in July 2006 [70]. The documented concentrations of GSM and MIB in 91% and 81% of the 67 and 64 studies, respectively, surpassed their respective OTCs of 4 and 15 ng·L^−1^. This indicates the widespread occurrence of these compounds and their potential impact on water quality.

In China, GSM concentrations have reached noteworthy peaks, including 1300 ng·L^−1^ in Chaohu Lake in August 2012 [33], 2711.5 ng·L^−1^ in Xionghe Lake in July 2007, 826.7 ng·L^−1^ in the same lake in November 2007 [66], and the aforementioned 7200 ng·L^−1^ in the YH Reservoir. Additionally, GSM concentrations exceeding 600 ng·L^−1^ have been documented in various countries, such as Germany, Japan, Korea, and the USA. MIB concentrations have also been reported at high levels in China, with Chaohu Lake recording a maximum concentration of 180.5 ng·L^−1^ in August 2012 [11]. Many other water bodies in China, including Dianchi Lake, Donghu Lake, Taihu Lake, Xionghe Reservoir, and a landscape lake in Beijing, have exhibited MIB values over 20 times the OTC. For example, MIB concentrations reached 450 ng·L^−1^ in Dianchi Lake in July 2003 [46], 317 ng·L^−1^ in Donghu Lake in January 1996 [56], 325 ng·L^−1^ in Taihu Lake in September 2009 [23], and 5302.7 ng·L^−1^ in a fishpond in July 2006 [70]. In other countries and regions, MIB concentrations generally ranged from 0 to 289 ng·L^−1^, except for Japan, where a concentration of 1400 ng·L^−1^ was reported [78]. These high concentrations of GSM and MIB not only indicate potential risks to water quality but also suggest the need for continued monitoring and management efforts to mitigate their impacts on drinking water supplies and aquatic ecosystems.

In contrast, both CYC and ION were reported in only 15 studies, encompassing seven water bodies (Table 3). Regarding CYC, it is noteworthy that in seven Chinese reservoirs [21] and the Llobregat River in Spain [81], CYC was not detected. However, the recorded peak values exceeded the OTC of 19.3 ng·L^−1^ in 14 publications, spanning seven water bodies. Specifically, in China, Eastern Chaohu Lake, a drinking water source, recorded concentrations as high as 800 ng·L^−1^ in August 2012 [33]. Western Chaohu Lake had a concentration of 105.6 ng·L^−1^ in January 2012 [11], while Eastern Chaohu Lake again reached 714.8 ng·L^−1^ in September 2013 [42]. Elevated concentrations were also reported in other Chinese lakes, including Dianchi Lake (450 ng·L^−1^ in September 2003 [46]), Taihu Lake (2080 ng·L^−1^ in September 2009 [23]), and landscape lakes in Beijing, reaching a remarkable 6000 ng·L^−1^ in May 2016 [45,69]. In different geographical regions, CYC has also been detected. For instance, Bao et al. (1997) reported concentrations ranging from 0 to 57 ng·L^−1^ in the Arno River in Japan in May 1995 [34], while Young et al. (1999) found 133 ng·L^−1^ in Winnebago Lake in the USA in August 1996 [40]. These findings highlight the widespread occurrence of CYC and the potential need for further investigation and monitoring in diverse aquatic systems.

Regarding ION, the reported maximum concentrations in 14 publications, inclusive of the current study, exceeded the odor threshold concentration (OTC) of 7 ng·L^−1^. Notably, ION was not detected in seven significant reservoirs located in eastern China, as reported by Wu et al. (2021b) [21], and concentrations in the Arno River in Italy ranged from 0 to 7 ng·L^−1^, as documented by Bao et al. (1997) [34]. Among the various water bodies studied, Chaohu Lake and Taihu Lake stand out as having been extensively investigated. Chaohu Lake recorded a peak concentration of 8300 ng·L^−1^ in December 2012 [33], while Taihu Lake exhibited a peak concentration of 1976.4 ng·L^−1^ in September 2017 [22]. These high concentrations indicate potential environmental concerns in these regions. In addition to Chaohu and Taihu lakes, other Chinese water bodies also showed elevated ION concentrations. For instance, Dianchi Lake recorded 570 ng·L^−1^ in September 2003 [46], Songhua Lake reached 8.9 ng·L^−1^ in September 2017 [22], and the main canal of the Middle Route of the South-to-North Water Diversion Project had concentrations with ranges of 11.973 ± 20.643 ng·L^−1^ between September 2018 and August 2019 [71]. Further, the Liangxi River had a concentration of 350 ng·L^−1^ in September 2018 [51], and raw water from a Chinese city registered a concentration of 358 ng·L^−1^ [26]. In contrast, limited data are available for ION concentrations in water bodies outside of China. This suggests that further research and monitoring efforts are needed to comprehensively understand the occurrence and impact of ION in global aquatic systems.

#### 2.4.2. Implications of Spatiotemporal Distribution of T/O Compounds for Management

Overall, numerous countries or regions worldwide grapple with profoundly severe T/O problems in their aquatic ecosystems. China has garnered extensive research attention due to its pronounced T/O challenges, which stand out as the most severe among studied regions. These challenges manifest not only in widespread distribution across diverse water bodies (e.g., lakes, reservoirs, rivers, canals, fishponds, and raw water from drinking water treatment plants) but also in the heightened concentrations of odoriferous substances they entail (see references in Table 3). The highest concentrations of these four T/O substances have been consistently reported in China. Furthermore, most documented water bodies exhibit maximum concentrations surpassing their corresponding OTCs. In other countries, there has been a relatively extensive amount of research on GSM and MIB. However, there has been comparatively less research on CYC and ION. Considering that the concentrations of both CYC and ION can reach 6000 and 8300 ng·L^−1^, respectively, in the field [33,45,69], it is advisable to include them in the scope of detection, as well (Table 4).

From the perspective of the temporal distribution of T/O compounds, most peak concentrations occur during the summer and autumn seasons. In terms of management and monitoring, it is crucial to recognize that many studies do not provide year-round coverage and tend to favor observations during these two seasons. Notably, certain T/O compounds in specific water bodies exhibit peak concentrations in other seasons, which recommended involving winter data in future predictions models ([96] and this study). Moreover, studies also emphasized the temporal variability of odor occurrences within the same water body across different years, which could be attributed to various factors, including environmental conditions, biological dynamics, water mixing patterns, and anthropogenic influences [11,22,23,33,35,42,64,65]. A comprehensive understanding of the temporal dynamics of odor events was crucial in developing water quality management and treatment strategies, facilitating the recognition of patterns and trends, and furnishing essential information for the establishment of early warning systems.

The spatial distribution of odorants reveals that odor issues were widespread in water bodies globally, with different water systems exhibiting diverse odorant scenarios. This variability could be attributed to factors such as nutrient concentration, sources, and geographical conditions. In China, lakes with severe eutrophication, such as Chaohu and Taihu Lake, experienced prominent odor problems. Among all the lakes in China, Taihu Lake stood out for its more severe T/O issues and increased attention, likely due to the warmer temperatures and well-developed economy in the surrounding areas [22]. Similarly, situated in the central part of China, Chaohu Lake is a vital managed water body grappling with significant eutrophication, demanding heightened attention. However, there is a noticeable dearth of literature addressing taste and odor occurrences in Chaohu Lake, highlighting the need for further research. For the same water body, such as Lake Taihu, variations in odor compounds among different regions exist. Deng et al. (2019) unveiled spatial disparities in nutrients and T/O compounds between blooming and non-blooming areas [64]. Elevated concentrations of compounds like CYC and ION in blooming areas contrasted with lower levels in non-blooming areas. Considering both the biological and non-biological factors in lake management facilitated the development of effective odor control strategies, thereby contributing to water quality management and ecological balance. Additionally, attention should be directed toward the distribution of odor compounds in different water layers. Research on Lake Zürich illuminated the significant seasonal dynamics of odorant concentrations influenced by lake stratification [86]. Internal factors, such as temperature gradients and nutrient distribution, led to distinct odorant concentrations across various water layers. Managing water extraction necessitated addressing odorant variability in specific lake layers, thereby influencing water quality at extraction points. Recognizing the importance of considering seasonal variations and lake stratification was crucial in water resource management for predicting and controlling odorant distribution, ensuring stable and safe water quality at intake points.

### 2.5. Sources and Influencing Factors of T/O Compounds in Freshwater Bodies of the World

Comprehending the sources and determining factors of T/O compounds is pivotal for preempting T/O challenges and laying the groundwork for their management. The variability in T/O compounds is shaped by a confluence of biological, physical, and chemical indicators (Table 4).

#### 2.5.1. Biotic Factors

To address odor issues in water bodies, it is crucial to study their sources and primary influencing factors. Typically, studies explored the relationship between biological factors and the concentration of odor substances to identify their producers. Biotic factors were generally assessed by examining the biomass or density of total cyanobacteria, specific genera and/or species, and various indices, including Chl-*a*, phycocyanin, and genes associated with T/O compounds synthesis.

The concentrations of GSM in various water bodies exhibit diverse correlations with Chl-*a* and other factors. Several studies have found positive correlations between GSM concentrations and Chl-*a*. For instance, Ma et al. (2013) observed this positive correlation in Taihu Lake, China [23], while Wu et al. (2021b) reported a similar trend in seven important reservoirs in eastern China [21]. Additionally, Smith et al. (2002) noted a positive correlation in Cheney Reservoir in the United States. However, contrasting results have been observed in other water bodies [14]. In the current study, no significant correlation was found between GSM concentrations and Chl-*a* in DECL. Similarly, Zhang et al. (2019) reported no significant correlation in Chaohu Lake and Songhua Lake in China, as well as in Taihu Lake, also in China [22]. Li et al. (2007) found no correlation in Dianchi Lake in China [46], Jahnichen et al. (2011) found no correlation in Wahnbach Reservoir in Germany [16], and Bruder et al. (2014) found no correlation in Eagle Creek Reservoir in the United States [49].

Similar variability was observed for the correlation between cyanobacteria and GSM concentrations. Positive correlations were found in some water bodies (e.g., Phayao Lake in Thailand [87], Bukhan River and Namhan River in South Korea [18], and Paldang Lake in Korea [15]), while in others (DECL (this study), Dianchi Lake in China [46], and Eagle Creek Reservoir in the US [49]), there was no apparent relationship between cyanobacteria and GSM. Notably, specific types of cyanobacteria, such as *Microcystis*, *Dolichospermum*, and *Pseudoanabaena*, have been extensively researched in relation to GSM. Positive correlations between GSM and *Microcystis* were observed in various water bodies, including Taihu Lake in China [23], California Aqueduct in the US [24], Bukhan River and Namhan River in South Korea [18], and Lake Winnebago in the US [40]. Moreover, in specific lakes, such as Western Chaohu Lake [42] and Dianchi Lake in China [46], Paldang Lake in Korea [82], Bukhan River and Namhan River in South Korea [18], raw water from drinking water treatment plants in Japan [78], and Diamond Valley Lake in the US [25], GSM showed positive correlations with *Dolichospermum*, while in others (Taihu Lake in China [65], nine sampling sites along the main canal of the Middle Route of South-to-North Water Diversion Project in China [71], San Vicente Reservoir [93] and Lake Skinner in the US [93], and Lake Y in Japan [80]), it exhibited a positive correlation with *Pseudoanabaena*. However, it is crucial to note that some studies report scenarios in which no correlation was observed. Furthermore, positive correlations between GSM and other factors, such as *Merismopedia* (Namhan River in South Korea [18]), *Phormidium* (Lake Kasumigaura in Japan [9]), *Planktothrix* (Eagle Creek Reservoir in the US [28]), *Cylindrospermopsis raciborskii* (Eagle Creek Reservoir in the US [49]), and *Coelosphaerium* (Lake Shinji in Japan [77]), have been reported in certain studies.

For MIB, its relationship with various factors mirrors that of GSM, displaying positive or non-correlative associations with Chl-*a*, cyanobacteria, *Microcystis*, *Pseudoanabaena*, *Oscillatoria*, *Planktothrix*, and diatoms. In certain studies, positive correlations have been established with phycocyanin in specific regions (seven important reservoirs in eastern China [21]), while no correlation was found with *Dolichospermum* (Dianchi Lake [46] and Lake Yangcheng in China [47]), or a negative correlation was reported (DECL (this study)). MIB exhibited no correlation with *Merismopedia* in certain contexts (Lake Yangcheng in China [47]). However, positive correlations have been reported with *Phormidium* in various water bodies (Huangpu River [58], Lake Yangcheng in China [47], raw water from drinking water treatment plants [78], and Lake Kasumigaura [9] and Lake Kasumigaura in Japan [17]), and a positive correlation has been observed with *Lyngbya* in a specific study (California Aqueduct in the US [24]).

In comparison, there has been relatively less extensive research on CYC and ION. Studies such as Taihu Lake [23,64] and Dianchi Lake in China [46] have shown a positive correlation between Chl-*a* and both CYC and ION. Additionally, in Liangxi River [51] and 13 eutrophic lakes in China [48], there was a positive association between Chl-*a* and CYC. However, in certain locations, including DECL (this study) and nine sampling sites along the main canal of the Middle Route of South-to-North Water Diversion Project in China [71], CYC did not exhibit a correlation with Chl-*a*. Similarly, in DECL (this study), Chaohu Lake [22], Songhua Lake [22], Taihu Lake [22], and nine sampling sites along the main canal of the Middle Route of South-to-North Water Diversion Project in China [71], ION was not correlated with Chl-*a*. Concerning cyanobacteria, a positive correlation with both CYC and ION was observed in Dianchi Lake in China [46], while in DECL (this study), there was no association. Specifically for *Microcystis*, studies such as seven landscape lakes in Beijing [45,69], Taihu Lake [23,64], Western Chaohu Lake [42], Eastern Chaohu Lake [33] and Dianchi Lake in China [46], and Lake Winnebago in the US [40] have shown a positive correlation between CYC and *Microcystis*, and ION was positively correlated with CYC in Taihu Lake [23,64] and Dianchi Lake in China [46]. However, in DECL (this study), both CYC and ION were unrelated to *Microcystis*. Furthermore, CYC and *Dolichospermum* exhibited no significant correlation, whereas ION demonstrated a positive correlation with *Dolichospermum* in this study. In Taihu Lake of China, both CYC and ION were unrelated to *Oscillatoria* [64].

It is worth noting that both GSM and MIB are often produced by bacteria. Therefore, some studies have explored their relationship with bacteria. For instance, in Phayao Lake in Thailand [87], Lake Kasumigaura in Japan [17], and Paldang Lake in Korea [82]), GSM exhibited a positive correlation with actinomycetes. It is noteworthy to mention that alternative findings have been reported by Lee et al. (2020) in the context of Paldang Lake in Korea [15], where the sampling period ranged from June to September 2021, as reported by Park et al. (2016) [82]. In this study, there was no discernible association observed between GSM and actinomycetes. In Eagle Creek Reservoir in the US [29], GSM was positively correlated with *α-Proteobacteria*. MIB showed a positive correlation with actinomycetes in Eagle Creek Reservoir in the US [28] and fishponds in China [70], while in Eagle Creek Reservoir in the US, MIB displayed a positive correlation with *Flavobacterium* [29].

A positive correlation between the copy number of the synthesis gene and T/O compounds has been identified in various studies, including those conducted in Paldang Lake in Korea [84], Qingcaosha Reservoir [61], and Lushui Reservoir in China [59]. Consequently, Cao et al. (2023) [62] proposed that the abundance of the MIB synthesis gene (mic) in both the DNA and RNA forms could serve as valuable parameters for early warning systems associated with MIB production.

The comprehensive findings revealed significant positive correlations among Chl-*a*, phycocyanin, cyanobacteria (*Microcystis*, *Dolichospermum*, *Merismopedia*, *Oscillatoria*, *Phormidium*, *Planktothrix*, *Pseudanabaena*, *Cylindrospermopsis*, *Coelosphaerium*, and *Lyngbya*), diatoms, and certain T/O compounds (Table 4). These identified biotic factors were implicated in the potential generation of T/O compounds in natural water bodies affected by algal blooms. As in some eutrophic lakes, cyanobacteria and T/O compounds, such as MIB and GSM, exhibited a significant positive correlation with Chl-*a*. This underscored that the nutritional status, particularly Chl-*a*, served as an excellent indicator for T/O compounds [14,97]. As a result, certain national regulatory authorities established Chl-*a* concentration standards to anticipate the occurrence of T/O issues in lakes [98]. However, other microorganisms, such as actinomycetes, *Flavobacterium*, and α-*Proteobacteria*, were also observed to be positively related to GSM or MIB, implying a possible source of T/O compounds [29]. In this case, the above biological indicators showed no apparent correlation with odorants, even in the same lake with different investigation periods or regions. There were also few articles reporting a positive correlation between phycocyanin and the synthesis gene with MIB, which has opened a new avenue for T/O exploration. However, relying solely on correlation analysis to trace the source may lead to misjudgments; therefore, further analysis, such as isolating and establishing cultures for direct determination, is essential to identify the exact algae species responsible for the production.

#### 2.5.2. Physical Factors

The occurrence of T/O compounds in water bodies exhibited a nuanced relationship with various physical indicators, as evidenced by diverse findings in multiple studies (Table 4). Temperature, a key factor, was shown to have both positive and negative correlations with the four T/O compounds. While positive correlations were observed in studies like Lake Taihu (for GSM, CYC, and particle-bound MIB and ION [64]) and seven important reservoirs in eastern China [21], instances of negative correlations, notably in Eagle Creek Reservoir [49] and Lake Taihu (for dissolved MIB [64]), challenged the generalization of these associations. Dissolved oxygen further complicated the picture, with instances of positive and negative correlations with T/O compounds across different studies, emphasizing the multifaceted nature of the interactions. Conductivity, water depth, turbidity, and total dissolved solids contributed additional dimensions to the complexity. For example, positive correlations between GSM and MIB with conductivity in a Chinese lake (DECL, this study) differed from findings in Eagle Creek Reservoir [49] and Feng-Shen Reservoir [57]. The negative correlation between MIB and water depth in East Taihu Lake introduced further variability [50]. The lack of correlation between GSM and turbidity in Paldang Lake underscored the site-specific nature of these relationships [15]. The inclusion of oxidation-reduction potential as a contributing factor in Eagle Creek Reservoir highlighted the need for a comprehensive understanding of redox conditions in T/O dynamics [49].

For other physical indicators, in DECL in China, GSM and MIB exhibit a positive correlation with conductivity (this study). Conversely, in Eagle Creek Reservoir in the US [49] and Feng-Shen Reservoir [57], GSM and MIB show no correlation with conductivity. Moreover, in DECL in China, CYC and ION are unrelated to conductivity. In Taihu Lake, MIB was negatively correlated with water depth [50]. In Paldang Lake in Korea, GSM was unrelated to turbidity [15]. Furthermore, in Eagle Creek Reservoir in the US, GSM and MIB displayed a positive correlation with total dissolved solids and showed no correlation with oxidation-reduction potential [49]. The intricate interplay between T/O compounds and physical indicators emphasized the importance of context-specific studies, recognizing the site-specific nature of these interactions for effective water quality management and odor control strategies.

#### 2.5.3. Nutrients

The relationships between T/O compounds and various nitrogen forms have been investigated across multiple studies. Although positive correlations have been identified in specific instances, such as the association between GSM and total nitrogen in seven important reservoirs in eastern China [21] and the correlations of MIB, CYC, and ION with total nitrogen in East Taihu Lake, China [44], as well as ION with total nitrogen in Liangxi River, China [51], the majority of studies conducted by Bruder et al. (2014) [49], Shi et al. (2023) [50], Yu et al. (2019) [44] and Zhang et al. (2019) [22], as well as the present investigation did not observe significant correlations. Furthermore, variations have been noted in the relationships with nitrate and ammonia nitrogen among different compounds across various studies. Specifically, for nitrate, GSM exhibited a positive correlation with it in DECL (this study), Songhua Lake [22], and Liangxi River in China [51], while no correlation was observed in Taihu Lake in China [22,44] or Eagle Creek Reservoir in the US [49].

MIB exhibited a positive correlation with nitrate in nine sampling sites along the main canal of the Middle Route of the South-to-North Water Diversion Project in China [71]. Conversely, no significant correlation was discerned in DECL (this study), Songhua Lake [22], Chaohu Lake [22], Taihu Lake in China [22], and Eagle Creek Reservoir in the US [49]. Similarly, CYC demonstrated a positive correlation with nitrate in the specified sampling sites [71], while no significant correlation was evident in the present study. In the case of ION, it displayed a positive correlation with nitrate in Songhua Lake [22] and nine sampling sites along the main canal of the Middle Route of the South-to-North Water Diversion Project in China [71]. Conversely, a negative correlation was observed in East Taihu Lake [44], and no significant correlation was found in DECL (this study), Chaohu Lake [22], or Taihu Lake in China [22]. Examining ammonia nitrogen, GSM demonstrated a positive correlation with it in Songhua Lake [22], while no significant correlation was observed in DECL (this study), Chaohu Lake [22] and Taihu Lake in China [22,44], or Eagle Creek Reservoir in the US [49]. MIB showed a positive correlation with ammonia nitrogen in DECL in China (this study) and Eagle Creek Reservoir in the US [49], a negative correlation in seven landscape lakes in Beijing [45,69], and no significant correlation in Songhua Lake, Chaohu Lake, Taihu Lake [22], and Feng-Shen Reservoir in China [57]. In this study, CYC was unrelated to ammonia nitrogen. Furthermore, ION exhibited a positive correlation with ammonia nitrogen in Songhua Lake [22] and Liangxi River in China [51], while no significant correlation was noted in DECL (this study), Chaohu Lake, or Taihu Lake in China [22]. Bruder et al. (2014) also investigated total Kjeldahl nitrogen, finding a positive correlation with GSM in Eagle Creek Reservoir in the US and no correlation with MIB [49].

In the investigation of odorants and total phosphorus, there was a lack of research on a positive correlation between GSM and total phosphorus. Conversely, in Chaohu Lake, GSM exhibited a negative correlation with total phosphorus [22]. In subsequent studies, namely, in DECL (this study), Taihu Lake in China [22,44], and Eagle Creek Reservoir in the US [49], no correlation was observed between GSM and total phosphorus. However, MIB showed a positive correlation with total phosphorus in Taihu Lake in China [23,50] and Eagle Creek Reservoir in the US [49] and a negative correlation in Lake Yangcheng, China [47]. In DECL (this study), Songhua Lake [22], Chaohu Lake [22], and Taihu Lake in China [22], MIB was unrelated to total phosphorus. CYC exhibited a positive correlation with total phosphorus in Western Chaohu Lake [42], Taihu Lake [23], and 13 eutrophic lakes in China [48] and was unrelated in DECL in China (this study). ION was positively correlated with total phosphorus in Western Chaohu Lake [42] and Taihu Lake in China [23], negatively correlated in DECL (this study) and Chaohu Lake [22], and unrelated in Songhua Lake [22] and Taihu Lake in China [22]. Regarding other forms of phosphorus, dissolved inorganic phosphorus was negatively correlated with GSM in Chaohu Lake, China [22]. Dissolved inorganic phosphorus and dissolved organic phosphorus were unrelated to GSM in Songhua Lake, and unrelated to MIB and ION in Songhua Lake and Chaohu Lake, China [22]. Concerning orthophosphate, GSM was positively correlated with it in Western Chaohu Lake, China [42], MIB was negatively correlated with it in seven landscape lakes in Beijing [45,69], and no correlation was found with the four odorants in DECL in China (this study). Furthermore, research explored the relationship between the N/P ratio and odorants. The N/P ratio was positively correlated with all four odorants in East Taihu Lake, China [44], and negatively correlated in Liangxi River [51], where MIB showed no correlation with the N/P ratio in Lake Yangcheng, China [47].

These comprehensive investigations delved into the intricate relationships between T/O compounds and various nitrogen and phosphorus forms in diverse water bodies (see references in Table 4). While specific instances illustrated positive correlations, such as the association between GSM and total nitrogen in certain Chinese reservoirs, the overall findings revealed considerable variability across studies. MIB, CYC, and ION exhibited diverse correlations with total nitrogen, emphasizing the complex nature of these interactions. Studies further unveiled nuanced relationships with nitrate and ammonia nitrogen, showcasing positive, negative, or non-significant correlations among different T/O compounds in different locations. Regarding phosphorus forms, the investigation uncovered intricate associations between T/O compounds and total phosphorus, with varying correlations observed for GSM, MIB, CYC, and ION across different water bodies. Additional exploration into other phosphorus forms and the nitrogen-to-phosphorus ratio highlighted the complexity of these relationships, displaying diverse trends in different contexts. The observed variability underscores the need for a contextual and location-specific approach to studying these relationships. Further research is essential to unravel the complexities and refine our comprehension of the factors influencing T/O compounds in aquatic ecosystems.

#### 2.5.4. Organic Matter

In various investigations, the association between odorants and organic matter has been examined using the following four primary indicators: UV_254_, specific UV absorbance (SUVA), potassium permanganate index (COD), and dissolved organic carbon (DOC). According to the research conducted by Zhang et al. (2019), it was observed that GSM and ION exhibit a negative correlation with UV_254_ in Songhua Lake, China [22]. However, in Chaohu Lake, China, GSM, MIB, and ION were found to be unrelated to UV_254_ [22]. Moreover, MIB demonstrated no correlation with UV_254_ in Songhua Lake [22]. Concerning SUVA, GSM was found to have no correlation with it in both Songhua Lake and Chaohu Lake [22]. On the other hand, MIB showed a positive correlation with SUVA in seven landscape lakes in Beijing [45,69] but no correlation in Songhua Lake, Chaohu Lake, or Taihu Lake in China [22]. ION displayed no correlation with SUVA in either Songhua Lake or Chaohu Lake [22]. Moving on to COD, GSM exhibited a positive correlation with it in Yuqiao Reservoir [74], while MIB demonstrated a positive correlation with COD in DECL in China (this study) and Yuqiao Reservoir [74]. In DECL in China, GSM, CYC, and ION were found to be unrelated to COD (this study). In terms of DOC, GSM displayed a negative correlation with it in Chaohu Lake [22] but no correlation in DECL in China (this study), Songhua Lake, or Taihu Lake [22]. MIB exhibited a positive correlation with DOC in DECL in China (this study), but a negative correlation in Songhua Lake and Chaohu Lake [22]. CYC and ION were unrelated to DOC in DECL in China, and ION showed no correlation with DOC in either Songhua Lake or Taihu Lake [22].

#### 2.5.5. Interrelationships among Metabolites

Regarding the interrelations among volatile organic compounds, both consistencies and variations were observed (Table 4). GSM demonstrated positive correlations with MIB in certain water bodies, such as Eagle Creek Reservoir in the US [49], Chaohu Lake [22], Lake Taihu [23], and East Taihu Lake in China [44] but exhibited a negative correlation in the DECL (this study). Additionally, GSM showed positive correlations with CYC and ION, presenting intricate associations in different aquatic environments.

The coexistence of odorants and toxins in aquatic environments represents a prevalent challenge encountered by numerous water systems; however, comprehensive research in this domain remains inadequate. The limited studies available found that GSM was negatively correlated with MC in DECL in China (this study). Conversely, in Eagle Creek Reservoir in the US [49], there was no discernible correlation between GSM and MC. Furthermore, in DECL in China (this study) and Eagle Creek Reservoir in the US [49], MIB demonstrated negative association with MC.

## 3. Conclusions

The comparative analysis of T/O occurrences in targeted lakes, considering various nutritional states and geographic locations, has proven to be an invaluable tool for early warning systems associated with algal blooms and T/O events. This study identified management solutions for recurrent off-flavor events, emphasizing the imperative to mitigate eutrophication in surrounding watersheds while acknowledging the potential impact of lake-wide mixing on off-flavor dynamics. Additionally, the research delved into the seasonal variation and complexity of odorant dynamics, highlighting disparities in concentration trends across lake regions and variations in intra- and extracellular concentrations of odorants. These intricacies posed challenges for regulatory and removal strategies, underscoring the importance of enhanced monitoring to comprehend concentration trends in water sources. Furthermore, the global impact of cyanobacterial outbreaks on lakes and reservoirs was acknowledged, resulting in odor-related issues affecting landscape ecology and drinking water safety. This study underscored the significance of fortifying research on the spatial and temporal variations of T/O compounds and their influencing factors to provide technical guidance for controlling cyanobacteria and ensuring the safety of drinking water. The results also provided insights into managing different ecotypes facing nuisance odorant problems, necessitating the development of methods to predict metabolite production for implementing appropriate water treatment procedures. A more profound understanding of the ecology of communities involved in T/O dynamics was deemed essential for establishing proactive management strategies based on key mechanisms driving T/O dynamics. Lastly, in shallow eutrophic lakes, this study recognized the complicated and dynamic environmental conditions, recommending the future consideration of environmental factors responsible for odorant removal processes, such as volatilization and hydrodynamic movement. The call for ongoing research efforts was emphasized, highlighting the necessity of advancing our understanding and developing targeted strategies for water quality management. Overall, these collective insights underscored the complexity of T/O dynamics in water bodies and emphasized the need for a comprehensive, interdisciplinary approach to effectively manage and mitigate associated issues.

Based on the research, the following recommendations were drawn: (1) Monitoring and Surveillance: Given the severe and widespread nature of T/O issues in freshwater ecosystems, especially in China, there is a need for continuous monitoring and surveillance of T/O compounds in drinking water sources. This should include monitoring for seasonal variations and across different water layers. (2) Seasonal Considerations: The temporal analysis revealed variations in odor occurrences within the same water body across different years, emphasizing the need to consider high T/O values in all seasons for water safety. This requires a seasonally adapted water quality management strategy. (3) Targeted Interventions: Since algae were identified as crucial contributors to odor compounds, targeted interventions are needed to control algae growth. This could include physical, chemical, or biological methods tailored to the specific water body and its environmental conditions. (4) Nutrient Management: Nutrients were likely to influence T/O issues, indicating the need for nutrient management strategies to reduce algae growth and subsequent T/O production. This could involve reducing nutrient inputs from agricultural runoff, industrial discharges, and urban wastewater. (5) Further Research and Validation: The correlation analysis alone was insufficient to provide definitive answers, emphasizing the need for further validation, such as algae isolation and analysis of odorant sources and properties. This will require more in-depth research to understand the mechanisms behind T/O production and identify effective control measures. (6) International Collaboration: Given the global nature of T/O issues, international collaboration and knowledge sharing are essential to develop comprehensive management strategies. This could involve sharing best practices, conducting joint research projects, and developing standardized monitoring and assessment protocols.

## 4. Material and Methods

### 4.1. Study Area Description and Hydrological Conditions of the Lake

**Figure 3:**
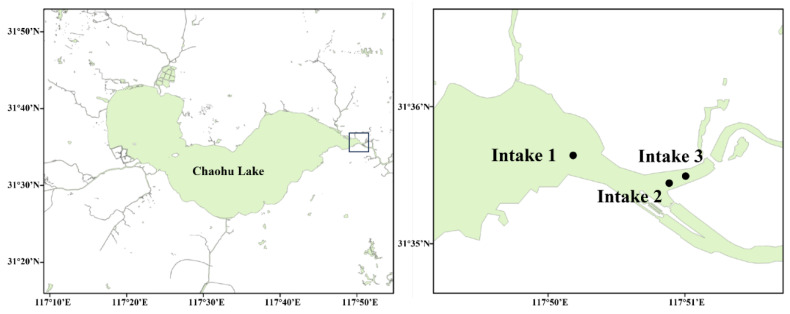
Sampling sites in the eastern drinking water source of Chaohu Lake.

With a surface area spanning 770 km^2^, a mean depth measuring 2.7 m, and a storage capacity reaching 2.1 billion m^3^, Chaohu Lake is a significant freshwater resource in the region. We selected the three primary drinking water intakes (117°50′15.13″ E/31°35′36.34″ N, 117°50′54.32″ E/31°35′24.82″ N, and 117°50′58.40″ E/31°35′32.30″ N, Figure 3) situated in the eastern sector of Chaohu Lake as the designated sampling sites, catering to a population exceeding 4 million consumers in the vicinity. Composite water samples were systematically collected monthly from September 2011 to August 2012, employing a 2.5 L sampler at 0.5 m intervals across the entire water column. Biological and physicochemical parameters and microcystins were analyzed parallel to the present work by Shang et al. (2015) and Shang et al. (2018) [11,52], with the minimum, maximum, and mean values detailed in Table 5.

### 4.2. Taste and Odor Compounds Analysis

#### 4.2.1. Chemicals, Material and Standards

High-performance liquid chromatography (HPLC)-grade methanol and sodium chloride (NaCl) of ensured quality were procured from Merck (Darmstadt, Germany) and Sigma-Aldrich Chemical Co. (St. Louis, MO, USA), respectively. Headspace bottles were acquired from Anpel Company (Shanghai, China), while ultrapure water was sourced from a Milli-Q water purification system (Millipore, Billerica, MA, USA). All other reagents adhered to guaranteed reagent-grade standards. The standards for T/O compounds, specifically, GSM and MIB from Sigma-Aldrich Chemical Co. and CYC and ION from Adamas Reagent Co., Ltd. (Basel, Switzerland), were employed. A stock solution of 1 mg·L^−1^, comprising the four target T/O compounds in methanol, was securely stored in darkness at −20 °C and subsequently diluted into aqueous working standard solutions before utilization.

#### 4.2.2. Sample Preparation and Analysis

The T/O compounds in samples were analyzed via solid-phase microextraction, combined with GC-MS, according to Watson et al. (2000) and Shang et al. (2018) [11,99]. In detail, one liter or more water samples were filtered immediately through a GF/C filter (Whatman, Dassel, Germany) at a low vacuum pressure. The dissolved fraction in water (80 mL) was transferred immediately into 125 mL headspace bottles with 25 g NaCl and a magnetic stir bar and measured using GC-MS in 12 h. Simultaneously, the particle-bound fraction retained on the filters was stored at −20 °C. For further extraction, the filters underwent three cycles of freeze-thawing and were then rinsed into a 125 mL vial with 80 mL of Milli-Q water. Subsequently, ultrasonication (JY92-2D, Xinzhi Co., Ningbo, China) was performed for 10 min in an ice bath. Enhanced recovery was achieved through the addition of NaCl and a magnetic stir bar. The T/O compound samples were stirred and extracted using a 2 cm-long CAR/DVB/PDMS fiber (Supelco, Inc., Bellefonte, PA, USA) at 65 °C for 30 min before injection into the GC-MS.

The T/O compounds, after extraction, underwent analysis utilizing an Agilent 6890 gas chromatograph interfaced with an Agilent 5973 mass spectrometer, equipped with a DB-5MS column (Agilent Technologies, Inc., Santa Clara, USA). The gas chromatography (GC) temperature program involved an initial holding phase at 60 °C for 2 min, followed by a gradual increase to 200 °C for 2 min at a rate of 8 °C/min, and subsequently elevated to 260 °C at a rate of 15 °C/min. Data analysis was executed in the selected ion monitoring mode. The identification of the targeted T/O compounds was accomplished through the comparison of retention times with established standards, alongside the verification of characteristic ions at m/z 112 and 126 for GSM, 107 and 95 for MIB, 137 and 152 for CYC, and m/z 177 and 91 for ION. The quantification of each analyte was carried out using the corresponding standard curve, with detection limits established at 0.2, 0.2, 0.5, and 0.4 ng·L^−1^ for GSM, MIB, CYC, and ION, respectively.

### 4.3. Literature Analysis

To acquire a comprehensive understanding, a literary analysis was undertaken to synthesize the exploration of T/O compounds in freshwater ecosystems globally, encompassing their concentrations and influencing factors. The database compilation involved meticulous keyword searches within the ISI Web of Science, encompassing 74 papers published from 1986 to 2023.

### 4.4. Statistical Analysis

The examination of concentrations for both dissolved and particulate T/O compounds was executed employing Origin 2018. A correlation analysis was conducted using the IBM Statistical Package for the Social Sciences Statistics 22. A redundancy analysis (RDA) was implemented through CANOCO 4.5 (Microcomputer Power, Ithaca, NY, USA) to ascertain the principal factors associated with T/O compounds [100].

## Figures and Tables

**Table 1:** Correlation coefficients between T/O compounds and phytoplankton and physicochemical parameters from September 2011 to August 2012 in the eastern drinking water source of Chaohu Lake.

Indexes	Factors	Geosmin	2-Methyl Isoborneol	*β*-Cyclocitral	*β*-Ionone
Biological	Chlorophyll-*a*	−0.133	0.300	0.206	0.095
	Cyanobacteria	−0.271	0.291	−0.013	0.052
	*Microcystis* spp.	−0.187	0.505 **	−0.039	−0.121
	*Dolichospermum* spp.	−0.418 *	−0.350 *	−0.087	0.387 *
Physical	Temperature	0.040	0.722 **	−0.066	−0.132
	pH	0.230	0.413 *	0.227	0.130
	Dissolved oxygen	0.004	−0.762 **	0.069	0.253
	Conductivity	0.379 *	0.788 **	0.250	−0.022
Nutrient	Total nitrogen	0.270	−0.010	−0.021	−0.071
	Nitrate	0.404 *	0.298	−0.014	−0.166
	Ammonia nitrogen	0.322	0.372 *	−0.025	−0.247
	Total phosphorus	−0.044	0.243	−0.096	−0.371 *
	Orthophosphate	−0.210	−0.155	−0.202	−0.188
Organic	Potassium permanganate index	0.210	0.452 **	0.287	−0.030
	Dissolved organic carbon	0.028	0.383 *	0.059	0.095

Note: Correlation significance is denoted with * *p* < 0.05 or ** *p* < 0.01.

**Table 2:** Correlation coefficients between concentrations of T/O compounds and microcystin congeners (MC-LR, MC-RR, and MC-YR) from September 2011 to August 2012 in the eastern drinking water source of Chaohu Lake.

	Geosmin	2-Methyl Isoborneol	*β*-Cyclocitral	*β*-Ionone	MC-LR	MC-RR	MC-YR
geosmin	1						
MIB	0.288	1					
*β*-cyclocitral	0.660 **	0.256	1				
*β*-ionone	0.408 *	0.169	0.654 **	1			
MC-LR	−0.183	0.228	0.239	0.014	1		
MC-RR	−0.136	0.217	0.157	0.077	0.802 **	1	
MC-YR	−0.368 *	0.128	−0.019	−0.265	0.817 **	0.643 **	1

Note: Correlation significance is denoted with * *p* < 0.05 or ** *p* < 0.01.

**Table 3:** Comparison of geosmin, 2-methyl isoborneol, *β*-cyclocitral, and *β*-ionone reported in different freshwater bodies of the world.

Country and Region	Sampling Site	Sampling Time	Geosmin (ng·L^−1^)	2-Methyl Isoborneol (ng·L^−1^)	*β*-Cyclocitral (ng·L^−1^)	*β*-Ionone (ng·L^−1^)	Reference
China	Eastern Chaohu Lake	August 2012, December 2012	0–1300 (August 2012)	0	0–800 (August 2012)	0–8300 (December 2012)	[33]
	Drinking water intakes in the Eastern Chaohu Lake	August 2011–August 2012	0.3–39.7 (March 2011)	0.1–180.5 (July 2012)	11.3–105.6 (January 2012)	8.5–136.6 (June 2012)	This study, [11]
	Western Chaohu Lake	July 2013–December 2013	0–28.3 (December 2013)	0.4–1.8 (August 2013)	0.7–714.8 (September 2013)	0.2–11.2 (September 2013)	[42]
	Chaohu Lake	September 2017	3.8–8.0 (September 2017)	0.5–9.5 (September 2017)		9.4–28.0 (September 2017)	[22]
	Dianchi Lake	June 2003–May 2004	0–130 (October 2003)	0–450 (July 2003)	20–450 (September 2003)	40–570 (September 2003)	[46]
	Donghu Lake	May 1995–April 1996	0–3.3 (June 1995)	10–317 (January 1996)			[56]
	Feng-Shen Reservoir	December 2000–July 2003		0–185 (July 2003)			[57]
	Huangpu River	January 2009–December 2009		0–71 (August 2009)			[58]
	Lushui Reservoir	July 2010–November 2011	7.2–45.3				[59]
	Miyun Reservoir	2009–2012		0–195 (September 2010)			[60]
	Qingcaosha Reservoir	May 2016–September 2016		0–7.3 (May 2016)			[61]
	QCS Reservoir	2021		0–99 (05/2021)			[62]
	Shenzhen Reservoir	October 28, 2016, May 8, 2017, 26/September 2017	1.2–4.2 (May 2017)	0			[63]
	Shiyan Reservoir	October 28, 2016, May 8, 2017, 26/September 2017	1.7–8.0 (May 2017)	22.1–52.9 (May 2017)			[63]
	Songhua Lake	September 2017	1.1–38.1 (September 2017)	0.6–3.4 (September 2017)		1.1–8.9 (September 2017)	[22]
	East Taihu Lake	September 2017	4.7–16.8 (September 2017)	13.1–32.7 (September 2017)	15.0–37.2	4.4–13.8 (September 2017)	[44]
		August 2020–November 2021		0.5–1446 (August 2021)			[50]
	Taihu Lake	January 2009–December 2009	0–4 (September 2009)	0–325 (September 2009)	5–2080 (September 2009)	32–573 (August 2009)	[23]
		June 2009–May 2010	0.2–4 (June 2009)	2–152 (July 2009)	1–360 (July 2009)	2–80 (September 2009)	[64]
		September 2017	0.3–1.2 (September 2017)	0.2–1.0 (September 2017)		2.2–1976.4 (September 2017)	[22]
		2020 published	1–20	15–100	22–530	8–600	[35]
			11.7–1446 (August 2021)				[65]
	Xionghe Reservoir	May 2007–April 2008	0–2711.5 (July 2007)	0–378.4 (April 2008)			[66]
		November 2007–October 2008	0–826.7 (November 2007)	0–148.1 (April 2008)			[67]
	FH Reservoir	July 2018–June 2019		3.0 ± 2.3–52.4 ± 8.4 (November 2019)			[68]
	Lake Yangcheng	2018–2019		0.4–940.6 (August 2018)			[47]
	Seven landscape lakes in Beijing	May 2016–September 2016		1–3509 (July 2016)	0–6000 (May 2016)		[45,69]
	Seven important reservoirs in eastern China	August 2019	13.11	18.39	0	0	[21]
	RW from eight cities	2005–2007	0–7200 (July 2007)	0–255 (September 2005)		0–358	[26]
	RW from rivers of DWTPs	November 2019–March 2010	0–6.1	0–25.8			[10]
	RW from lake/reservoir of DWTPs	November 2019–March 2010	0–9.1	0–65.0			[10]
	RW of 56 DWTPs in 31 major cities	July 2011–December 2011	0–5.5	0–104			[43]
	Fishponds	April 2006–January 2007	0.3–12.1 (July 2006)	0.5–5302.7 (July 2006)			[70]
	MRSNWDP	September 2018–August 2019		7.431 ± 9.631	12.371 ± 12.800	11.973 ± 20.643	[71]
	LWSP	March 2016–December 2017	5.15–17.66 (Winter)				[72]
	Liangxi River	September 2018	3–10	30–119	18–239	50–350	[51]
	Nanping Reservoir	2017–2020		0–113 (August 2020)			[73]
	Zhuxiandong Reservoir	March–Apirl 2021		51.7 ± 12.8 (Apirl 2021)			[73]
	Yuqiao Reservoir	2018–2021	0–193 (August 2018)	0–938.30			[74]
	13 eutrophic lakes in China	May 2018–September 2019			0–49.05 (September 2019)		[48]
Egypt	Solar Lake	March 1997, June 1997, July 1997, August 1997	–	–			[75]
Germany	Schleinsee Lake	1984,1985	0–180 (July 1984)				[76]
	Wahnbach Reservoir	May–October 2006–2009	0–600 (August)	0			[16]
Italy	Arno River	May 8, 1995	15–70 (May 1995)	0	0–57 (May 1995)	0–7 (May 1995)	[34]
Japan	Lake Kasumigaura	September 1989–October 1990	0–90 (September 1989)	0–150 (October 1990)			[9]
		1994–1997	0–560 (March 1995)	0–185 (April 1995)			[17]
	Lake Shinji	September 2009–October 2009	3–640 (October 2009)				[77]
	RW of DWTPs	October 2010–September 2012	0–520	0–1400			[78]
	A reservoir in Mie Prefecture	December 10th, 2019	0–9.53				[79]
	Lake Y in Nagasaki Prefecture	March–July 2022	<2	0–13			[80]
Spain	Llobregat River	1997–1999	5–200	10–20	0		[81]
Korea	Paldang Lake	June 2012–September 2012 April 2017–November 2018	0–4384 (July 2012) 0–246 (August 2017) 2–31 (July 2020)	0–22 (July 2012) 0–280 (October 2017)			[15,82,83,84]
	North Han River and Han River	December 2011	2–1640				[85]
	Bukhan River	2011–2015	0–810 (August 2014)				[18]
	Namhan River	2011–2015	0–75 (September 2015)				[18]
Switzerland	Lake Zürich	1995–1996	3.1–23 (December 1995)				[86]
Thailand	Phayao Lake	June 2012–February 2013	0–12.0 (October 2012)	0.1–1.1			[87]
North America	Eastern Ontario Lake and the Upper Saint Lawrence River	1996–1997	5–20 (September 1996)	2–25 (September 1996)			[88]
		July 1998, September 1998	0–20 (September 1998)	0–60 (September 1998)			[89]
	Ontario Lake	2007 published	0–200 (August–September)				[90]
	Saint Lawrence River	2000–2001	8–60	0–26			[19]
		2007 published	10–60	10–60			[90]
Wales, U.K.	Nine reservoirs	July 2019–August 2020	0.3–420	0.57–58			[91]
United States of America	California Aqueduct	August 1990–October 1990	3–48 (August 1990)				[24]
		July 1991–November 1991		0–78			[24]
	Cheney Reservoir	August 1999–October 2000	0–37 (July 2000)	0–2			[14]
		May 2001–June 2015	1–113 (July)				[20]
	Diamond Valley Lake	May 2000	0–750 (May 2000)				[25]
		October 2004	2–63 (October 2004)				[25]
	Eagle Creek Reservoir	2008–2010	0–109.4 (October 2009)	0–223.7 (May 2010)			[49]
		January 2013–December 2013	0–77.3 (May 2013)	0–111.8 (May 2013)			[28,29]
	McDaniel Lake	1983–2002	0–33	0–90			[92]
	San Vicente Reservoir	1996	23				[93]
	Winnebago Lake	August 20, 1996	18 (August 1996)		133 (August 1996)		[40]
	Three water supply reservoirs	1999–2002	0–2 (September 2000)	0–46 (September 2000)			[41]
	Four Reservoirs	May 2001–December 2012	0–133	0–224			[94]
	Drinking water Reservoir	November 2013–December 2013, 06/2014–August 2014		10–289 (August 2014)			[27,95]

Note: DWTPs: drinking water treatment plants; RW: raw water; MRSNWDP = nine sampling sites along the main canal of the Middle Route of South-to-North Water Diversion Project; LWSP = local water supply plant, Shanghai, China.

**Table 4:** Influencing factors to geosmin, 2-methyl isoborneol, *β*-cyclocitral, and *β*-ionone in different freshwater bodies of the world.

		Geosmin	2-Methyl Isoborneol	*β*-Cyclocitral	*β*-Ionone
CHL	+	TL–CN–January 2009–December 2009 [23] CR–US–August 1999–October 2000 [14] REC–CN–August 2019 [21]	TL–CN–June 2009–May 2010 [64] TL–CN–January 2009–December 2009 [23] REC–CN–August 2019 [21] ETL–CN–August 2020–November 2021 [50] LR–CN–2022 [51]	TL–CN–June 2009–May 2010 [64] TL–CN–January 2009–December 2009 [23] DL–CN–June 2002–May 2003 [46] LR–CN–2022 [51] TEL–CN–05/18–09/19 [48]	TL–CN–June 2009–May 2010 [64] TL–CN–January 2009–December 2009 [23] DL–CN–June 2002–May 2003 [46]
	#	DECL–CN–September 2011–August 2012 (This study) SL–CN–September 2017 [22] CL–CN–September 2017 [22] TL–CN–September 2017 [22] TL–CN–June 2009–May 2010 [64] DL–CN–June 2002–May 2003 [46] WR–DE–May–October 2006–2009 [16] ER–US–2008–2010 [49]	DECL–CN–September 2011–August 2012 (This study) SL–CN–September 2017 [22] CL–CN–September 2017 [22] TL–CN–September 2017 [22] DL–CN–June 2002–May 2003 [46] ER–US–2008–2010 [49]	DECL–CN–September 2011–August 2012 (This study) SWDP–CN–September 2018–August 2019 [71]	DECL–CN–September 2011–August 2012 (This study) SL–CN–September 2017 [22] CL–CN–September 2017 [22] TL–CN–September 2017 [22] SWDP–CN–September 2018–August 2019 [71]
CYA	+	PL–TH–June 2012–February 2013 [87] BR–KR–2011–2015 [18] NR–KR–2011–2015 [18] PAL–KO–June 2012–September 2012 [15]	LL–CN–May–September 2016 [45,69] DWR–US–November 2013–December 2013 [27] YC–CN–2018–2019 [47] ETL–CN–August 2020–November 2021 [50]	DL–CN–June 2002–May 2003 [46]	DL–CN–June 2002–May 2003 [46]
	#	DECL–CN–September 2011–August 2012 (This study) DL–CN–June 2002–May 2003 [46] ER–US–2008–2010 [49]	DECL–CN–September 2011–August 2012 (This study) DL–CN–June 2002–May 2003 [46] ER–US–2008–2010 [49]	DECL–CN–September 2011–August 2012 (This study)	DECL–CN–September 2011–August 2012 (This study)
PHY	+		REC–CN–August 2019 [21]		
MIC	+	TL–CN–January 2009–December 2009 [23] CA–US–August 1990 [24] BR–KR–2011–2015 [18] NR–KR–2011–2015 [18] LW–US–August 20, 1996 [40]	DECL–CN–September 2011–August 2012 (This study) TL–CN–June 2009–May 2010 [64] TL–CN–January 2009–December 2009 [23] YC–CN–2018–2019 [47]	LL–CN–May–September 2016 [45,69] TL–CN–June 2009–May 2010 [64] WCL–CN–December 2013 [42] ECL–August 2012, December 2012 [33] TL–CN–January 2009–December 2009 [23] DL–CN–June 2002–May 2003 [46] LW–US–August 20, 1996 [40]	TL–CN–June 2009–May 2010 [64] TL–CN–January 2009–December 2009 [23] DL–CN–June 2002–May 2003 [46]
	#	DECL–CN–September 2011–August 2012 (This study) TL–CN–June 2009–May 2010 [64] DL–CN–June 2002–May 2003 [46]	DL–CN–June 2002–May 2003 [46]	DECL–CN–September 2011–August 2012 (This study)	DECL–CN–September 2011–August 2012 (This study)
DOL	+	PAL–KO–June 2012–September 2012 [82] WCL–CN–December 2013 [42] RW–JP–October 2010–September 2012 [78] DL–CN–June 2002–May 2003 [46] BR–KR–2011–2015 [18] NR–KR–2011–2015 [18] Diamond Valley Lake in the US [25]			DECL–CN–September 2011–August 2012 (This study)
	–	DECL–CN–September 2011–August 2012 (This study)	DECL–CN–September 2011–August 2012 (This study)		
	#		DL–CN–June 2002–May 2003 [46] YC–CN–2018–2019 [47]	DECL–CN–September 2011–August 2012 (This study)	
MER	+	NR–KR–2011–2015 [18]			
	#		YC–CN–2018–2019 [47]		
OSC	+		TL–CN–June 2009–May 2010 [64] RW–JP–October 2010–September 2012 [78]		
	#	TL–CN–June 2009–May 2010 [64]	YC–CN–2018–2019 [47]	TL–CN–June 2009–May 2010 [64]	TL–CN–June 2009–May 2010 [64]
PHO	+	LK–JP–September 1989–1990.10 [9]	RW–JP–October 2010–September 2012 [78] HR–CN–January 2009–December 2009 [58] LK–JP–September 1989–October 1990 [9] LK–JP–1994–1997 [17] YC–CN–2018–2019 [47]		
PLA	+	ECR–US–January 2013–December 2013 [28] ER–US–2008–2010 [49]	MR–CN–2009–2012 [60] DL–CN–June 2002–May 2003 [46] ER–US–2008–2010 [49] NP–CN–2017–2020 [73]		
	#		YC–CN–2018–2019 [47]		
PSE	+	SVR–US–1996 [93] LSK–US–1996 [93] LY–JP–2022 [80] TL–CN–2021 [65] SWDP–CN–September 2018–August 2019 [71]	SR–CN–April–July 2018 [63] QR–CN–May 2016–September 2016 [61] SWDP–CN–September 2018–August 2019 [71] ETL–CN–August 2020–November 2021 [50] NP–CN–2017–2020 [73] YQR-CN–2018–2021 [74]		
	#	ER–US–2008–2010 [49]	ER–US–2008–2010 [49]		
CYL	+	ER–US–2008–2010 [49]			
COE	+	LS–JP–September–October 2009 [77]			
LYN	+		CA–US–August 1990 [24]		
DIA	+		DWR–US–November 2013–December 2013 [27]		
	#		ER–US–2008–2010 [49]		
ACT	+	PL–TH–June 2012–February 2013 [87] LK–JP–1994–1997 [17] PAL–KO–June 2012–September 2012 [82]	ECR–US–January 2013–December 2013 [28] FP–CN–April 2006–January 2007 [70]		
	#	PAL–KO–June 2012–September 2012 [15]			
FLA	+		ECR–US–January 2013–December 2013 [29]		
PRO	+	ECR–US–January 2013–December 2013 [29]			
GEN	+	PAL–KO–07–10/2020 [84]	QR–CN–May 2016–September 2016 [61] LSR–CN–July 2010–November 2011 [59] QCS Reservoir–CN–2021 [62] PAL–KO–July–October 2020 [84]		
T	+	TL–CN–June 2009–May 2010 [64] REC–CN–August 2019 [21] YQR–CN–2018–2021 [74]	DECL–CN–September 2011–August 2012 (This study) TL–CN–June 2009–May 2010 for p–MIB [64] FR–CN–December 2000–July 2003 [57] YC–CN–2018–2019 [47]	TL–CN–June 2009–May 2010 [64] TEL–CN–05/18–09/19 [48]	TL–CN–June 2009–May 2010 for p–ION [64]
	–	ER–US–2008–2010 [49]	TL–CN–June 2009–May 2010 for d–MIB [64]	DECL–CN–September 2011–August 2012 (This study)	TL–CN–June 2009–May 2010 for d–ION [64]
	#	DECL–CN–September 2011–August 2012 (This study)	ER–US–2008–2010 [49] August 2012	DECL–CN–September 2011–August 2012 (This study)	DECL–CN–September 2011–August 2012 (This study)
pH	+		DECL–CN–September 2011–August 2012 (This study) LL–CN–May 2016–09 [45,69]		
	#	DECL–CN–September 2011–August 2012 (This study) ER–US–2008–2010 [49]	FR–CN–December 2000–July 2003 [57] ER–US–2008–2010 [49]	DECL–CN–September 2011–August 2012 (This study)	DECL–CN–September 2011–August 2012 (This study)
DO	+	REC–CN–August 2019 [21]	TL–CN–June 2009–May 2010 [64]		TL–CN–June 2009–May 2010 for d–ION [64]
	−	SL–CN–September 2017 [22] TL–CN–June 2009–May 2010 [64] August 2012	DECL–CN–September 2011–August 2012 (This study)	TL–CN–June 2009–May 2010 [64]	SL–CN–September 2017 [22] TL–CN–June 2009–May 2010 for p–ION [64]
	#	DECL–CN–September 2011–August 2012 (This study) TL–CN–September 2017 [22] ER–US–2008–2010 [49]	SL–CN–September 2017 [22] CL–CN–September 2017 [22] TL–CN–September 2017 [22] ER–US–2008–2010 [49]	DECL–CN–September 2011–August 2012 (This study)	DECL–CN–September 2011–August 2012 (This study) CL–CN–September 2017 [22] TL–CN–September 2017 [22]
CON	+	DECL–CN–September 2011–August 2012 (This study)	DECL–CN–September 2011–August 2012 (This study)		
	#	ER–US–2008–2010 [49]	FR–CN–December 2000–July 2003 c ER–US–2008–2010 [49]	DECL–CN–September 2011–August 2012 (This study)	DECL–CN–September 2011–August 2012 (This study)
WD	+		ETL–CN–August 2020–November 2021 [50]		
TUR	#	PAL–KO–June 2012–September 2012 [15]			
TDS	+	ER–US–2008–2010 [49]	ER–US–2008–2010 [49]		
ORP	#	ER–US–2008–2010 [49]	ER–US–2008–2010 [49]		
TN	+	REC–CN–August 2019 [21]	TL–CN–September 2017 [44]	TL–CN–September 2017 [44]	TL–CN–September 2017 [44] LR–CN–2022 [51]
	#	DECL–CN–September 2011–August 2012 (This study) SL–CN–September 2017 [22] CL–CN–September 2017 [22] TL–CN–September 2017 [22] ETL–CN–September 2017 [44] ER–US–2008–2010 [49]	DECL–CN–September 2011–August 2012 (This study) SL–CN–September 2017 [22] CL–CN–September 2017 [22] TL–CN–September 2017 [22] ER–US–2008–2010 [49] ETL–CN–August 2020–November 2021 [50]	DECL–CN–September 2011–August 2012 (This study)	DECL–CN–September 2011–August 2012 (This study) SL–CN–September 2017 [22] CL–CN–September 2017 [22] TL–CN–September 2017 [22]
NO_3_	+	DECL–CN–September 2011–August 2012 (This study) SL–CN–September 2017 [22] LR–CN–2022 [51]	SWDP–CN–September 2018–August 2019 [71]	SWDP–CN–September 2018–August 2019 [71]	SL–CN–September 2017 [22] SWDP–CN–September 2018–August 2019 [71]
	−				ETL–CN–September 2017 [44]
	#	TL–CN–September 2017 [22] ETL–CN–September 2017 [44] ER–US–2008–2010 [49]	DECL–CN–September 2011–August 2012 (This study) SL–CN–September 2017 [22] CL–CN–September 2017 [22] TL–CN–September 2017 [22] ER–US–2008–2010 [49]	DECL–CN–September 2011–August 2012 (This study)	DECL–CN–September 2011–August 2012 (This study) CL–CN–September 2017 [22] TL–CN–September 2017 [22]
TKN	+	ER–US–2008–2010 [49]			
	#		ER–US–2008–2010 [49]		
NH_4_	+	SL–CN–September 2017 [22]	DECL–CN–September 2011–August 2012 (This study) ER–US–2008–2010 [49]		SL–CN–September 2017 [22] LR–CN–2022 [51]
	−		LL–CN–May-September 2016 [45,69]		
	#	DECL–CN–September 2011–August 2012 (This study) CL–CN–September 2017 [22] TL–CN–September 2017 [22] ETL–CN–September 2017 [44] ER–US–2008–2010 [49]	SL–CN–September 2017 [22] CL–CN–September 2017 [22] TL–CN–September 2017 [22] FR–CN–December 2000–July 2003 [57]	DECL–CN–September 2011–August 2012 (This study)	DECL–CN–September 2011–August 2012 (This study) CL–CN–September 2017 [22] TL–CN–September 2017 [22]
TP	+		TL–CN–January 2009–December 2009 [23] ER–US–2008–2010 [49] ETL–CN–August 2020–November 2021 [50]	WCL–CN–December 2013 [42] TL–CN–January 2009–December 2009 [23] TEL–CN–05/18–09/19 [48]	WCL–CN–December 2013 [42] TL–CN–January 2009–December 2009 [23]
	−	CL–CN–September 2017 [22]	YC–CN–2018–2019 [47]		DECL–CN–September 2011–August 2012 (This study) CL–CN–September 2017 [22]
	#	DECL–CN–September 2011–August 2012 (This study) SL–CN–September 2017 [22] TL–CN–September 2017 [22] ETL–CN–September 2017 [44] ER–US–2008–2010 [49]	DECL–CN–September 2011–August 2012 (This study) SL–CN–September 2017 [22] CL–CN–September 2017 [22] TL–CN–September 2017 [22]	DECL–CN–September 2011–August 2012 (This study)	SL–CN–September 2017 [22] TL–CN–September 2017 [22]
DIP	−	CL–CN–September 2017 [22]			
	#	SL–CN–September 2017 [22]	SL–CN–September 2017 [22] CL–CN–September 2017 [22]		SL–CN–September 2017 [22] CL–CN–September 2017 [22]
DOP	#	SL–CN–September 2017 [22]	SL–CN–September 2017 [22] CL–CN–September 2017 [22]		SL–CN–September 2017 [22] CL–CN–September 2017 [22]
PO_4_	+	WCL–CN–December 2013 [42]			WCL–CN–December 2013 [42]
	−		LL–CN–May–September 2016 [45,69]		
	#	DECL–CN–September 2011–August 2012 (This study)	DECL–CN–September 2011–August 2012 (This study)	DECL–CN–September 2011–August 2012 (This study)	DECL–CN–September 2011–August 2012 (This study)
N/P	+	ETL–CN–September 2017 [44]	ETL–CN–September 2017 [44]	ETL–CN–September 2017 [44]	ETL–CN–September 2017 [44] LR–CN–2022 [51]
	#		YC–CN–2018–2019 [47]		
UV_254_	+				
	−	SL–CN–September 2017 [22]			SL–CN–September 2017 [22]
	#	CL–CN–September 2017 [22]	SL–CN–September 2017 [22] CL–CN–September 2017 [22]		CL–CN–September 2017 [22]
SUVA	+		LL–CN–May–September 2016 [45,69]		
	−				SL–CN–September 2017 [22]
	#	SL–CN–September 2017 [22] CL–CN–September 2017 [22]	SL–CN–September 2017 [22] CL–CN–September 2017 [22] TL–CN–September 2017 [22]		CL–CN–September 2017 [22]
COD	+	YQR–CN–2018–2021 [74]	DECL–CN–September 2011–August 2012 (This study) YQR–CN–2018–2021 [74]		
	#	DECL–CN–September 2011–August 2012 (This study)		DECL–CN–September 2011–August 2012 (This study)	DECL–CN–September 2011–August 2012 (This study)
DOC	+		DECL–CN–September 2011–August 2012 (This study)		
	−	CL–CN–September 2017 [22]	SL–CN–September 2017 [22] CL–CN–September 2017 [22]		CL–CN–September 2017 [22]
	#	DECL–CN–September 2011–August 2012 (This study) SL–CN–September 2017 [22] TL–CN–September 2017 [22]		DECL–CN–September 2011–August 2012 (This study)	DECL–CN–September 2011–August 2012 (This study) SL–CN–September 2017 [22] TL–CN–September 2017 [22]
MC	+	August 2012	August 2012		
	−	DECL–CN–September 2011–August 2012 (This study)	DECL–CN–September 2011–August 2012 (This study) ER–US–2008–2010 [49]		
	#	ER–US–2008–2010 [49]			
GSM	+		ER–US–2008–2010 [49] CL–CN–September 2017 [22] TL–CN–January 2009–December 2009 [23] ETL–CN–September 2017 [44]	TL–CN–2020 published [35] TL–CN–January 2009–December 2009 [23] ETL–CN–September 2017 [44]	TL–CN–2020 published [35] CL–CN–September 2017 [22] SL–CN–September 2017 [22] TL–CN–January 2009–December 2009 [23] ETL–CN–September 2017 [44]
	−		DECL–CN–September 2011–August 2012 (This study)		
	#		TL–CN–2020 published [35] SL–CN–September 2017 [22] TL–CN–September 2017 [22]		TL–CN–September 2017 [22]
MIB	+	ER–US–2008–2010 [49] CL–CN–September 2017 [22] TL–CN–January 2009–December 2009 [23] ETL–CN–September 2017 [44]		TL–CN–2020 published [35] TL–CN–January 2009–December 2009 [23] ETL–CN–September 2017 [44]	TL–CN–2020 published [35] CL–CN–September 2017 [22] TL–CN–January 2009–December 2009 [23] ETL–CN–September 2017 [44]
	−	DECL–CN–September 2011–August 2012 (This study)			
	#	TL–CN–2020 published [35] SL–CN–September 2017 [22] TL–CN–September 2017 [22]			SL–CN–September 2017 [22] TL–CN–September 2017 [22]
CYC	+	TL–CN–2020 published [35] TL–CN–January 2009–December 2009 [23] ETL–CN–September 2017 [44]	TL–CN–2020 published [35] TL–CN–January 2009–December 2009 [23] ETL–CN–September 2017 [44]		TL–CN–2020 published [35] TL–CN–January 2009–December 2009 [23] ETL–CN–September 2017 [44]
ION	+	TL–CN–2020 published [35] SL–CN–September 2017 [22] CL–CN–September 2017 [22] TL–CN–January 2009–December 2009 [23] ETL–CN–September 2017 [44]	TL–CN–2020 published [35] CL–CN–September 2017 [22] TL–CN–January 2009–December 2009 [23] ETL–CN–September 2017 [44]	TL–CN–2020 published [35] TL–CN–January 2009–December 2009 [23] ETL–CN–September 2017 [44]	
	#	TL–CN–September 2017 [22]	SL–CN–September 2017 [22] TL–CN–September 2017 [22]		

Note: +: There is a positive correlation between T/O compound and each indicator. #: There is no correlation between the T/O compound and each indicator. −: There is a negative correlation between T/O compound and each indicator. CHL = chlorophyll-*a*; CYA = cyanobacteria; PHY = phycocyanin; MIC = *Microcystis*; DOL = *Dolichospermum*; MER = *Merismopedia*; OSC = *Oscillatoria*; PHO = *Phormidium*; PLA = *Planktothrix*; PSE = *Pseudanabaena*; CYL = *Cylindrospermopsis raciborskii*; COE = *Coelosphaerium*; LYN = *Lyngbya*; DIA = Diatom; ACT = actinomycetes; FLA = *Flavobacterium*; PRO = α-proteobacteria; GEN = 2-methyl isoborneol synthesis gene; T = temperature; DO = dissolved oxygen; CON = conductivity; WD = water depth; TUR = turbidity; TDS = total dissolved solids; ORP = oxidation-reduction potential; TN = total nitrogen; NO_3_ = nitrate; TKN = total Kjeldahl nitrogen; NH_4_ = ammonia nitrogen; TP = total phosphorus; DIP: dissolved inorganic phosphate; DOP: dissolved organic phosphate; PO_4_ = orthophosphate; N/P = N/P ratio; UV = UV_254_; SUVA = specific UV absorbance; COD = potassium permanganate index; DOC = dissolved organic carbon; MC = microcystin; GSM = geosmin; MIB = 2-methyl isoborneol; CYC = *β*-cyclocitral; ION = *β*-ionone; CN = China; DE = Germany; JP = Japan; KO = Korea; KR = South Korea; TH = Thailand; US = United States; DECL = drinking water intakes in Eastern Chaohu Lake [this study]; CL = Chaohu Lake; ECL = Eastern Chaohu Lake; WCL = Western Chaohu Lake; TL = Taihu Lake; ETL = East Taihu Lake; SL = Songhua Lake; DL = Dianchi Lake; DVL = Diamond Valley Lake; PAL = Paldang Lake; PL = Phayao Lake; LL = seven landscape lakes in Beijing; LS = Lake Shinji; LSK = Lake Skinner; LK = Lake Kasumigaura; LY = Lake Y; LW= Lake Winnebago; YC = Lake Yangcheng; TEL = 13 eutrophic lakes in China; LR = Liangxi River; CR = Cheney Reservoir; ECR = Eagle Creek Reservoir; SVR = San Vicente Reservoir; SR = Shiyan Reservoir; SWDP = nine sampling sites along the main canal of the Middle Route of South-to-North Water Diversion Project; QCS = QCS Reservoir; NP = Nanping Reservoir; QR = Qingcaosha Reservoir; MR = Miyun Reservoir; WR = Wahnbach Reservoir; LSR = Lushui Reservoir; FR = Feng-Shen Reservoir; YQR = Yuqiao Reservoir; REC = seven important reservoirs in eastern China; DWR = drinking water reservoir; FH = FH Reservoir; BR = Bukhan River; HR = Huangpu River; NR = Namhan River; CA = California Aqueduct; RW = raw water from drinking water treatment plants; FP = fishponds.

**Table 5:** Minimum, maximum, and average values of biological, physicochemical indices, and microcystins observed in the eastern drinking water source of Chaohu Lake from September 2011 to August 2012.

Indexes	Minimum	Maximum	Mean
Chlorophyll-*a* (µg·L^−1^)	5.3	75.6	25.7
Cyanobacterial density (×10^4^ cells·mL^−1^)	0.1	21.9	9.4
*Microcystis* spp. density (×10^4^ cells·mL^−1^)	0.0	18.5	5.6
*Dolichospermum* spp. density (×10^4^ cells·mL^−1^)	0.1	17.1	3.7
Temperature (°C)	6	31	19
pH	7.3	8.8	8.1
Dissolved oxygen (mg·L^−1^)	6.0	12.4	9.4
Conductivity (μS·cm^−1^)	293	416	329
Total nitrogen (mg·L^−1^)	0.48	3.29	1.67
Nitrate (mg·L^−1^)	0.09	1.46	0.59
Ammonia nitrogen (mg·L^−1^)	0.06	0.38	0.19
Total phosphorus (mg·L^−1^)	0.02	0.19	0.08
Orthophosphate (mg·L^−1^)	0.00	0.02	0.00
Potassium permanganate index (mg·L^−1^)	3.8	8.3	5.5
Dissolved organic carbon (mg·L^−1^)	3.71	5.82	4.22
MC-LR (μg·L^−1^)	0.07	1.90	0.53
MC-RR (μg·L^−1^)	0.12	3.73	0.79
MC-YR (μg·L^−1^)	0.00	4.18	0.82

## Data Availability

No datasets were generated or analyzed during the current study.
